
JOURNAL INFORMATION
==============================
NLM Title Abbreviation: Inter Econ
Journal ID: Inter Econ
NLM Journal ID: 101086399

Inter Economics

ISSN: 0020-5346

ARTICLE INFORMATION
==============================
PMCID: PMC7110261
DOI: 10.1007/s10272-020-0872-6
Article ID: 872
Article version: 1
Subjects: Forum Introduction

The Cost of Growing Older: Challenges for European Pension Systems

Electronic publication date: 2020 Apr 1
Print publication date: 2020
Volume: 55
Issue: 2
First page: 68
Last page: 68
Copyright: © The Author(s) 2020
License: Open Access This article is licensed under a Creative Commons Attribution 4.0 International License, which permits use, sharing, adaptation, distribution and reproduction in any medium or format, as long as you give appropriate credit to the original author(s) and the source, provide a link to the Creative Commons licence, and indicate if changes were made.
The images or other third party material in this article are included in the article’s Creative Commons licence, unless indicated otherwise in a credit line to the material. If material is not included in the article’s Creative Commons licence and your intended use is not permitted by statutory regulation or exceeds the permitted use, you will need to obtain permission directly from the copyright holder.
To view a copy of this licence, visit http://creativecommons.org/licenses/by/4.0/.
License URL: https://creativecommons.org/licenses/by/4.0/

issue-copyright-statement: © The Author(s) 2020

==============================
DOI: 10.1007/s10272-020-0872-6

### The Cost of Growing Older: Challenges for European Pension Systems

**Pension reform has been on the agendas of many European policymakers for the better part of the last three decades. While some EU countries have made sweeping reforms several years ago, others are currently in the process. Undoubtedly, most will need to re-evaluate their systems due to the coronavirus crisis, at least temporarily. With Europe's ageing populations, declining fertility rates and increasing life expectancy, the associated rise in the old-age dependency ratio puts strain on unfunded, pay-as-you-go pension systems. This implicit pension debt has important macroeconomic implications. Often politically controversial and subject to intense policy debate, pension reforms may reduce entitlements for some demographic groups of the population. This has lead to widespread public dissatisfaction among the affected groups. It is necessary to look at a country's history and key features of its pension system in order to understand the related policy discussion. This Forum analyses the effects of different pension arrangements – with a focus on the challenges, history and demographics of Finland, France, Germany and Italy – on labour markets, on national growth, and on the distribution of burdens and benefits.**

### Pension Systems in the EU – Some Policy Issues

**Mikkel Barslund**, Centre for European Policy Studies, Brussels, Belgium.

### Lessons From Italy: A Good Pension System Needs an Effective Broader Social Policy Framework

**Daniele Franco**, Bank of Italy, Rome, Italy.
**Pietro Tommasino**, Bank of Italy, Rome, Italy.

### Towards a Universal Pension Points System in France

**Hervé Boulhol**, Organisation for Economic Co-operation and Development, Paris, France.

### They Will Definitely Need Us, When We Are 64: Old-Age Provision in Germany

**Martin Werding**, Ruhr-University Bochum; and CESifo Research Network, Munich, Germany.

### The Finnish Pension System and Its Future Challenges

**Tarmo Valkonen**, ETLA Economic Research, Helsinki, Finland.
